# AI-Based Electromyographic Analysis of Single-Leg Landing for Injury Risk Prediction in Taekwondo Athletes

**DOI:** 10.3390/healthcare14030292

**Published:** 2026-01-23

**Authors:** Jun-Sik Kim, Fatima Faridoon, Jaeyeop Choi, Junghwan Oh, Juhyun Kang, Hae Gyun Lim

**Affiliations:** 1 Daejeon Gwanjeo High School, Daejeon 35358, Republic of Korea; 2 Industry 4.0 Convergence Bionics Engineering, Pukyong National University, Busan 48513, Republic of Korea; 3 Smart Gym-Based Translational Research Center for Active Senior’s Healthcare, Pukyong National University, Busan 48513, Republic of Korea; 4 Department of Biomedical Engineering, Pukyong National University, Busan 48513, Republic of Korea

**Keywords:** Taekwondo, single-leg landing, electromyography, artificial intelligence, muscle activation pattern

## Abstract

**Background/Objectives**: Improper landing mechanics in Taekwondo can lead to non-contact injuries such as ankle sprains and knee ligament tears, highlighting the necessity for objective methods to evaluate landing stability and injury risk. Electromyography (EMG) enables the examination of muscle activation patterns; however, conventional analyses based on simple averages have limited predictive value. **Methods**: This study analyzed EMG signals recorded during single-leg landings (45 cm height) in 30 elite male Taekwondo athletes. Participants were divided into regular exercise groups (REG, n = 15) and non-exercise groups (NEG, n = 15). Signals were segmented into two phases. Eight features were extracted per muscle per phase. Classification models (Random Forest, XGBoost, Logistic Regression, Voting Classifier) were used to classify between groups, while regression models (Ridge, Random Forest, XGBoost) predicted continuous muscle activation changes as injury risk indicators. **Results**: The Random Forest Classifier achieved an accuracy of 0.8365 and an F1-score of 0.8547. For regression, Ridge Regression indicated high performance (R^2^ = 0.9974, MAE = 0.2620, RMSE = 0.4284, 5-fold CV MAE: 0.2459 ± 0.0270), demonstrating strong linear correlations between EMG features and outcomes. **Conclusions:** The AI-enabled EMG analysis can be used as an objective measure of the study of the individual landing stability and risk of injury in Taekwondo athletes, but its clinical application has to be validated in the future by biomechanical injury indicators and prospective cohort studies.

## 1. Introduction

Taekwondo is a combat sport that requires substantial lower-limb strength, precise body control, and repetitive execution of dynamic kicking and landing techniques. During both training and competition, athletes frequently perform jumping and spinning maneuvers that depend on effective neuromuscular coordination [1]. Among these, single-leg landing is one of the most critical movements. The ability to stabilize the body and absorb impact after executing turning or spinning kicks affects not only athletic performance but also the likelihood of injury [2,3,4]. Improper landing control such as insufficient knee flexion, excessive inversion, or delayed muscle activation can alter load distribution and joint alignment, leading to non-contact injuries [5,6]. Epidemiological studies have reported that ankle sprains, anterior cruciate ligament (ACL) tears, and muscle strains are among the most common injuries in Taekwondo, accounting for a substantial proportion of time-loss events [7,8,9]. Because many of these injuries result from neuromuscular control deficits rather than direct collisions, understanding the neuromuscular factors that determine landing stability is essential for injury prevention.

Previous biomechanical studies have long focused on quantifying parameters that influence safe landing performance. Ground reaction force, displacement of the center of mass, joint flexion angles, and muscle activation levels have been identified as key indicators of landing stability and load absorption [4,10]. Among these factors, surface electromyography (EMG) provides direct information on muscle activation by recording the electrical signals produced during contraction [11,12]. EMG enables quantitative assessment of muscle recruitment, activation timing, and coordination among synergistic and antagonistic muscle groups. In Taekwondo, EMG offers a valuable means to examine how lower-limb muscles contribute to balance and shock attenuation during single-leg landing.

Single-leg landing stability has been traditionally evaluated through multiple biomechanical methods that include joint kinematics, ground reaction force analysis, and dynamic postural control metrics [13,14,15,16]. Previous studies examined limb asymmetries that demonstrated the bilateral strength imbalances can affect landing mechanics and injury risk [16]. Furthermore, recent research on mini-trampoline training showed the protective effects on leg stiffness and reactive power during landing tasks [13,14]. Differential training approaches have similarly proved improvements in vertical jump technique and bilateral coordination, which highlights the importance of integrating many biomechanical parameters that include limb asymmetries, joint loading strategies, and dynamic postural control incomprehensive assessments of landing stability [15]. These findings highlight that landing stability appears from complex interactions among multiple biomechanical factors, requiring analytical approaches capable of capturing such multivariate relationships.

However, EMG data are inherently complex with activation patterns that vary across muscles and movement phases due to individual characteristics and task-specific conditions. Traditional statistical methods, such as *t*-tests and linear regression, evaluate variables independently and assume linear relationships, which limits their ability to capture the non-linear interactions within coordinated movement. Previous Taekwondo studies have explored single parameters, such as mean or peak EMG activity, providing only limited insight into neuromuscular control strategies [17].

Artificial intelligence (AI) offers a framework for addressing these analytical limitations. Machine learning algorithms, including Random Forest and XGBoost, can model complex and non-linear relationships among variables while handling multiple features simultaneously [18,19,20,21,22,23]. Unlike conventional statistics that analyze each factor independently, AI-based models can integrate EMG-derived features, such as mean activation levels across multiple muscles and phases, to identify multivariate patterns associated with landing stability and performance variability. This capability enables more comprehensive interpretation of how combinations of muscle activities contribute to stability, even when only summary features rather than continuous time-series signals are available.

The application of AI to EMG analysis provides additional methodological advantages. It allows simultaneous evaluation of interdependence among muscles that act together during landing, offering a more realistic representation of movement. AI-based models integrate EMG-derived features such as mean activation levels across multiple muscles and phases to identify patterns correlated with landing stability and performance variability. Furthermore, explainable AI techniques such as permutation-based feature importance and Shapley Additive Explanations (SHAP) identify specific muscles that strongly influence AI models outputs, improving interpretability and linking computational results with biomechanical mechanisms relevant to training and rehabilitation [24,25].

Despite these advantages, AI-based EMG analysis has been used in gait analysis, general athletic performance monitoring, and injury prediction in team sports [18,19,21] but has been infrequently applied to martial arts landing mechanics sports, such as Taekwondo. Previous Taekwondo research has been based primarily on descriptive statistics, or univariate comparisons of separate EMG parameters, which are not able to reflect combined patterns of muscle coordination between stable and unstable motion. Notably, the current literature has gaps: (1) multi-muscle models of landing stability based on EMG traits, (2) biomechanical load injury risk regression models that can quantify the biomechanical load during single-leg landing, and (3) explainable AI integration that may define neuromuscular patterns with the strongest predictors of landing outcomes. Due to this, the association between multivariate EMG-derived measures, dynamic postural control, and risk of injury during Taekwondo-specific landing tasks is not well understood.

In response to this need, the present study analyzed EMG signals recorded during single-leg landings of Taekwondo athletes using AI-based classification and regression models. EMG data were collected from nine major lower-limb muscles of elite male athletes and summarized as normalized mean and standard deviation features for each landing phase. Classification models were developed to distinguish between stable and unstable landings, while regression models predicted continuous injury risk indices derived from biomechanical load parameters. By combining both approaches, the study aimed to provide categorical and quantitative assessments of landing stability. Featureanalysis was further conducted to determine which muscles and EMG characteristics most significantly influenced model performance. Through this integrative approach, the study seeks to establish an objective analytical framework for evaluating neuromuscular control and predicting injury risk in Taekwondo athletes.

## 2. Materials and Methods

### 2.1. Participants

This study was conducted with 30 elite male Taekwondo athletes from universities and high schools. Our study included only male athletes to control for well-documented sex-related differences in neuromuscular control strategies, lower-limb biomechanics, landing mechanics, and muscle activation patterns [21,25]. Previous research has demonstrated that males and females exhibit significantly different muscle recruitment strategies during landing tasks, with females showing greater quadriceps dominance, reduced hamstring activation, and altered knee valgus patterns [25]. All participants had accumulated over five years of Taekwondo training experience and had no history of lower-limb injuries within six months before the experiment. The participants had an average age of 19.3 ± 2.1 years, height of 174.5 ± 5.8 cm, and weight of 68.2 ± 7.4 kg, respectively. Participants were divided into two groups: regular exercise group (REG, n = 15), who completed a 12-week Get Set injury prevention program with progressive intensity across three phases (3+ sessions/week, 15 min per session), and non-exercise group (NEG, n = 15), who maintained their standard Taekwondo training without additional injury prevention exercises. Before participating, all subjects were fully informed about the study’s purpose, procedures, and potential risks, and voluntarily provided written informed consent.

**Figure 1:**
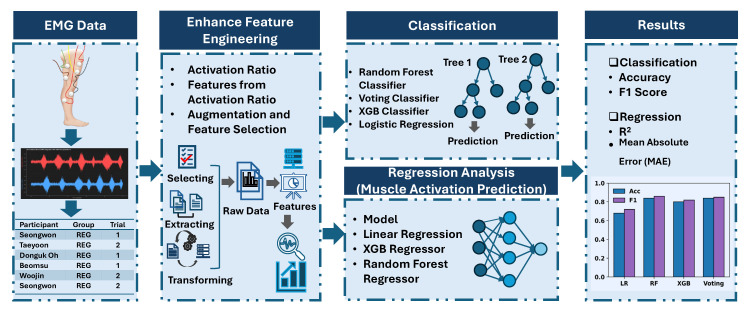
Workflow of the analysis of single-leg landing for injury risk prediction in Taekwondo athletes.

Figure 1 shows the methodology workflow of our study from data acquisition to Result analysis.

### 2.2. Experimental Procedure and Data Collection

The single-leg landing motion was measured to simulate the landing phase following a roundhouse kick, one of Taekwondo’s representative offensive techniques. Participants performed a drop-landing task by jumping off a 45 cm-high box and landing on one leg. EMG signals were recorded at 1000 Hz using the Ultium ESP wireless system (Noraxon, Scottsdale, AZ, USA) with surface electrodes on nine lower-limb muscles following SENIAM guidelines [12]. Raw signals were pre-processed using MyoResearch™ (MR 4.0.124, Vicon Plug-in v1.0.3.12) software s with band-pass filtering (20–450 Hz), followed by full-wave rectification and RMS smoothing to create amplitude envelopes. Signals were normalized using the peak dynamic method (dividing by maximum activation per participant across trials), which is appropriate for ballistic landing movements.

In this study, we developed a systematic approach to transform the pre-processed EMG features into a meaningful pattern by using an enhanced feature engineering pipeline, which ensures optimal feature extraction and preparation for subsequent machine learning applications.

#### 2.2.1. Feature Extraction and Transformation

Following the data collection phase, the next phase was the feature extraction and transformation, which is necessary for extracting optimized and meaningful features from the complex EMG data. For this, we calculated basic metrics such as the mean absolute value, root mean square (RMS), combined magnitude, non-linear relationships such as P1_squared, P2_squared, and average magnitude. This method ensures a thorough description of unseen features.

#### 2.2.2. EMG Signal Processing and Feature Extraction

Following the data collection phase, the next phase was the feature extraction and transformation, which is necessary for deriving informative and optimized features from the complex EMG data. Landing was segmented into two phases: Phase 1 (P1) from initial contact to peak vertical ground reaction force (impact absorption), and Phase 2 (P2) from peak force to maximum knee flexion (postural stabilization). For each muscle in each phase, we extracted eight features:

(1) $$P1\;P2\;Mean=\frac{\left(P1+P2\right)}{2}$$

(2) $$P1_{Squared}={\left(P1\right)}^{2},P2_{Squared}={\left(P2\right)}^{2}$$

(3) Log_P1=log(1+P1),Log_P2=log(1+P2)

(4) Log_Ratio=log(P1/(P2+ε))

(5) Activation_Ratio=P1/(P2+ε)

(6) $$Activation\_Magnitude=\surd (P1^{2}+P2^{2})$$

(7) Activation_Distance=|P1−P2|

(8) Activation_Change_Percent=(P2−P1)/(P1+ε)×100

#### 2.2.3. Data Augmentation and Feature Optimization

Our methodology used a dual strategy to prepare the dataset for strong predictive modeling. It expands the data through augmentation and refines the features through selection. To make the model more reliable and reduce performance issues from class imbalance in the training data for the classification task (groups: REG and NEG), we applied stratified 5-fold cross-validation to ensure balanced class representation in all training and testing splits. We used a feature-level data augmentation strategy, and we applied the Synthetic Minority Oversampling Technique (SMOTE) to overcome the class imbalance on the extracted features of the summary (MAV, RMS, ratios). This is justified by many factors: first, summary statistics represent physiologically significant ranges, making the interpolation between similar patterns of biomechanically plausible; second, the k nearest neighbor algorithm of SMOTE (k = 5) constrains artificial generation of samples to the local neighborhood of measured values, thus preventing the generation of physiologically impossible combinations; third, SMOTE has been validated in EMG-based classification for gait and sports biomechanics [26], which makes performance estimations conservative, and only reflective of real samples. Every feature was z-score standardized before using SMOTE. SMOTE creates synthetic examples of the minority class, as determined by an automatic sampling strategy, by working directly in the feature space.

### 2.3. Model Training and Evaluation

The process then moved to model training after feature extraction and data augmentation, which included two tasks: classification of each group and regression of muscle activation changes.

#### 2.3.1. Classification Model Training

For the classification task of predicting the skilled group, we trained the model on pre-processed feature data. To evaluate the discriminative power of the newly engineered features, all classifiers were deliberately configured with highly restrictive hyperparameters. Logistic Regression (LR) was trained under a very strict L2 (regularization of a model) with a C set to 0.005. C is a regularization parameter used to control how a model handles misclassification. This penalty reduces the magnitude of feature coefficients, compelling the model to use only the most vital and distinct features for classification. The Random Forest (RF) model was employed on 200 decision trees (n_estimators = 200) without depth restrictions, allowing trees to grow until leaves were pure or contained a minimum number of samples. This configuration enables the ensemble to capture complex, non-linear interactions among features while maintaining generalization through bootstrap aggregation and random feature subsampling at each split. The XGBoost model was configured with 200 boosting rounds (n_estimators = 200) and a maximum tree depth of 3 (max_depth = 3), and a learning rate of 0.1. The depth limitation of 3 prevents overfitting while allowing sufficient model complexity to capture feature interactions up to three-way combinations, which is appropriate for our feature set and sample size.

#### 2.3.2. Regression Model Training and Tuning

Regression models were applied to predict muscle activation changes and were tuned to maximize performance on the standardized features set. The Ridge Regression (RR) utilized L2 regularization controlled by the alpha parameter (α = 1.0) to prevent excessively large coefficient values. This approach provided an optimal balance between model stability and data fitting, thereby enhancing the model’s ability to generalize to unseen data. The RF regression model was implemented with 200 trees (n_estimators = 200) with no depth restrictions, allowing the model to capture complex and non-linear relationships among the features, which is an essential capability for accurately predicting continuous variables such as muscle activation change. XGBoost regression employed an advanced gradient boosting framework and was fine-tuned to maximize predictive accuracy for muscle activation change estimation. The model was trained with 200 trees (n_estimators = 200) and a learning rate of 0.1, which not only improved accuracy but also strengthened generalization and reduced the risk of overfitting. XGBoost’s sequential ensemble approach builds trees that correct errors made by previous trees, potentially offering superior predictive performance for non-linear patterns in EMG data.

### 2.4. Evaluation Metrics

For the classification task, the evaluation metrics focused on stability, balance, and accuracy. The evaluation metrics used for the classification task were test accuracy, 5-fold cross-validation (CV) accuracy, recall, and the F1-score. For the regression task, the R^2^ score, mean absolute error (MAE), and root mean squared error (RMSE) were used. The R^2^ score indicates how well the model fits the observed values, MAE represents the average absolute deviation, and RMSE reflects the average magnitude of prediction errors. The process then moved to model training after feature extraction and data augmentation, which included two tasks: classification of each group and regression of muscle activation changes.

(9) Accuracy=(TP+TN)/(TP+TN+FP+FN)

(10) Recall=TP/(TP+FN)

(11) F1=2×(Precision×Recall)/(Precision+Recall)

(12) Where Precision=TP/(TP+FP)

(13) $$R^{2}=1-\left(\frac{SS_{res}}{SS_{tot}}\right)$$

(14) MAE=(1/n)×Σ|y_i−ŷ_i|

(15) $$RMSE=\surd [(1/n)\times \Sigma (y\_i-ŷ\_i{)}^{2}]$$

TP (true positives), TN (true negatives), FP (false positives), and FN (false negatives) represent correct and incorrect classifications, while for regression SS_res = Σ(y_i − ŷ_i)^2^, SS_tot = Σ(y_i − ȳ)^2^, y_i represents actual values, ŷ_i represents predicted values, ȳ is the mean of actual values, and n is the number of samples.

As presented in Table 1, different machine learning models were used with certain hyperparameters to maximize performance. The parameter n_estimators defines how many decision trees to use in the ensemble models (200 in all RF and XGBoost models) to avoid overfitting by reducing the complexity of any single decision tree. max depth is used to control the depth of each decision tree (max depth = 3 in all XGBoost models) to prevent overfitting by limiting the model’s complexity. The learning rate (0.1) determines the contribution of each tree to the final prediction, balancing learning speed and accuracy. eval metric = logloss measures the accuracy of probability predictions in a classification task, random state = 42 controls randomness to ensure reproducibility, and voting= soft in the Voting Classifier averages predicted probabilities across multiple models to achieve better performance.

## 3. Results

### 3.1. Experimental Setup

The machine learning models were developed using Python version 3.13 in Jupyter Notebook version 7.3.2 within the Anaconda environment. The computational setup was equipped with an AMD Ryzen 9 9950X 16-core processor running at 4.30 GHz and 64 GB of RAM.

### 3.2. Data Integrity and Feature Basis

Before applying the machine learning models, the data was cleaned and made robust. The features, including the feature engineering (P1 and P2) and categorical variables (groups), were validated to ensure no missing data. This ideal data integrity eliminated the need for imputation and ensured that subsequent feature engineering and model training operated on a robust feature foundation. The direct input features, P1 and P2, were used to construct all other higher-order and ratio-based analytical features, which were applied in both the classification and regression tasks.

### 3.3. Classification Task Performance

The classification models that are tested on the pre-processed data achieved strong results, as shown in Table 2.

The Random Forest Classifier achieved the best performance among all classification models, achieving a test accuracy of 0.8365 and an F1-score of 0.8547. Its stability and reliable generalization have been supported by a 5-fold CV accuracy of 0.7980 ± 0.0422. The Voting Classifier achieved an accuracy of 0.8269, the XGB Classifier achieved an accuracy of 0.7981, with a 5-fold CV accuracy of 0.7691 ± 0.0360, and the Logistic Regression showed the lowest performance with an accuracy of 0.6729.

**Figure 2:**
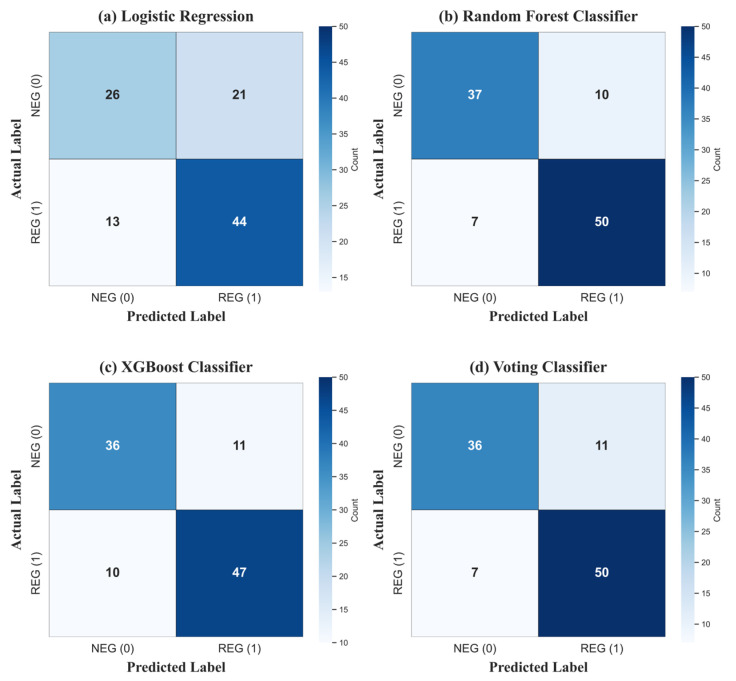
Confusion matrix for four classification models showing true negatives (top-left), false positives (top-right), false negatives (bottom-left), and true positives (bottom-right). Color intensity indicates prediction frequency. (**a**) Logistic Regression, (**b**) Random Forest Classifier, (**c**) XGB Classifier, and (**d**) Voting Classifier. Random Forest achieved the highest true positive rate (50) with minimal false negatives (7).

The four classification models were evaluated using confusion matrices, as shown in Figure 2. The Random Forest and the Voting Classifier achieved the highest scores, resulting in 50 true positives and a low number of false negatives (7). The XGBoost classifier performed comparably, whereas Logistic Regression was the weakest, producing a high number of false positives (21) and the fewest true negatives (26).

### 3.4. Regression Task Performance

The Regression models achieved effective results as shown in Table 3.

**Figure 3:**
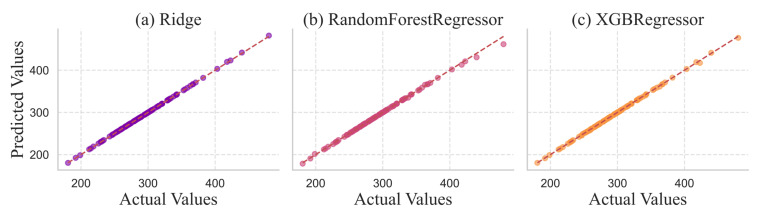
Predicted versus actual values for regression models. Each point represents one prediction, with the diagonal line indicating perfect prediction (predicted = actual). Tighter clustering around the diagonal indicates better model performance.

RR models demonstrated an almost perfect R^2^ score of 0.9999, which means that the relationship between engineered features and the target variable is very linear. Moreover, Ridge provided the lowest error values: the MAE is 0.2620, and the RMSE is 0.4284. The 5-fold CV MAE (0.2459) made it even more stable (0.0270). Though non-linear models (XGB and RF) also achieved high scores of R^2^ of 0.9997 and 0.9982, respectively. The Random Forest Regressor produced the largest RMSE value (2.1562), indicating that it was most susceptible to large individual prediction errors. Figure 3 shows prediction of actual values vs. predicted values for the regression models.

## 4. Discussion

**Figure 4:**
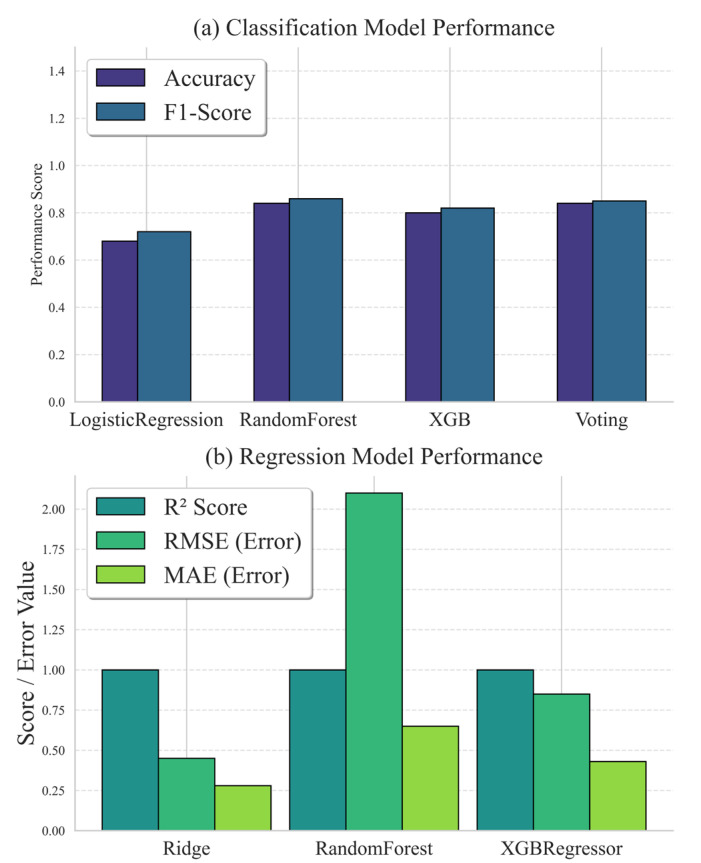
Comparison of both classification and regression models in terms of evaluation. (**a**) Classification model and (**b**) regression model.

This research focused on developing an AI-based EMG analysis of single-leg landings for injury risk prediction in Taekwondo athletes and then comparing the results of four well-known classification algorithms, which included LR, RF, XGBoost, and Voting Classifier, as well as three regression models to predict injury risk and classify landing stability in Taekwondo athletes. For the main classification task (skilled vs. unskilled), the ensemble methods were more effective. The best performance was obtained by the Random Forest Classifier with an F1-score of 0.8547 and a test accuracy of 0.8365. Conversely, the weakest model was the linear Logistic Regression, which achieved an accuracy of 0.6731, consistent with its inability to capture the complex, non-linear neuromuscular interactions involved in human movement. Figure 4a shows the comparison of the classification models. Our observation that the Random Forest outperformed the other classifiers in identifying subtle movement patterns aligns with the findings of a previous study, which achieved an accuracy of 0.987 in identifying various lower-limb movement patterns using the Random Forest. This further validates the algorithm’s strong performance in processing time and frequency-domain EMG features [27].

However, the pattern was different in the regression task that predicted the change in continuous muscle activation, which is an index of injury risk, interestingly. The RR model demonstrated an almost perfect fit to the data, with an R^2^ score of 0.999 and an MAE of 0.262. This performance strongly indicates that the relationship between the engineered EMG features (P1/P2 values, activation ratios) and the continuous target variable is highly linear. This linearity rendered complex non-linear models unnecessary. Although the RF and XGBoost Regressors achieved high R^2^ values, they did not outperform the simpler RR model. Figure 4b shows the performance comparison of the regression models.

This methodological framework, based on improved feature engineering and AI classification, is supported by related literature in combat sports (Table 4). Notably, the most important methodological decision was the deliberate simplification of the classification models, such as setting the maximum tree depth to 3 for the RF. This limitation was imposed not to achieve the best possible performance but to test the inherent discriminative power of the newly engineered features and activation ratios. This feature-driven focus is also consistent with a previous study that demonstrated that feature selection (Sequential Backward Selection) could substantially improve SVM classification for muscle performance characterization of athletes, with feature quality being oftentimes more significant than model complexity [26]. Moreover, our use of RMS-derived and ratio-based features is important because, as reported in a previous study, there are significant variations in the values of maximal EMG depending on the signal processing methodologies (including RMS) and thus it is essential to choose a robust signal processing methodology when handling ballistic movements such as those in Taekwondo [28].

Lastly, the correlation between muscle activity and Taekwondo actions is important. Previous authors reported large disparities in integrated EMG during the retraction phase of a roundhouse kick between ‘hit’ and ‘miss’ actions, especially in the rectus femoris or tibialis anterior [30]. By investigating the landing phase of a single leg after the kick, our study logically extends this prior work. By analyzing the coordinated activity of nine lower-limb muscles, we were able to predict the resulting injury risk, as the post-kick stabilization processes were directly correlated with quantitative outcome measures in our study.

The current study demonstrates that machine learning models can accurately predict activation_change_percent from engineered EMG features, validating our feature engineering methodology. However, this does not establish construct validity for ‘injury risk’ prediction, as no biomechanical injury risk markers or prospective injury outcomes were measured. The relationship between activation_change_percent and actual injury incidence remains unvalidated. Future research must: (1) collect validated biomechanical injury risk metrics (e.g., knee valgus angles > 10°, vertical loading rates > 100 BW/s, asymmetry indices > 15%) alongside EMG data, (2) conduct prospective cohort studies tracking athletes over competitive seasons to record actual injury occurrences, and (3) validate whether EMG-derived predictions correlate with observed injury rates. Only through such validation can clinical injury prediction utility be established.

Overall, the positive results obtained with the RF Classifier and the exceptionally high accuracy of the RR model demonstrate that AI-based analysis of EMG features provides a two-pronged analytical framework for assessing landing stability and injury risk in Taekwondo athletes. These findings have significant practical implications for trainers and physical therapists. Although these are promising outcomes, future studies should focus on model explainability. With the introduction of model-agnostic tools such as SHAP, it will be possible to identify which muscles or activity patterns drive the model’s predictive decisions. Moreover, these predictive models should be externally validated on a larger and more heterogeneous group of athletes to ensure their generalizability.

Although this paper has shown that machine learning may be effectively used to estimate activation_change_percent using engineered EMG features, multiple limitations need to be addressed to put these results into perspective. To begin with, data is only based on young male black belts only (N = 30), and the findings might not be applicable to female practitioners and those of other levels of skill. Second, the use of EMG summative statistics with no incorporation of kinematic or kinetic measures (i.e., ground reaction forces) is another limitation. Moreover, we recognize that the exceptionally good regression accuracy is mainly justified by the mathematical consistency of our feature engineering framework and not its direct clinical performance. Since the regression target is derived from a mathematical measure, and not a validated biomechanical measure (e.g., knee valgus) or a clinical outcome measure, the current version of the model has high precision in capturing training-induced changes in neuromuscular measures, but not yet in actual injury incidence. To reduce this gap, future studies should shift their focus to longitudinal designs of study instead of continued cross-sectional observations. By monitoring athletes over a long duration, one will be able to correlate a particular change in the patterns of activation with recorded clinical improvements. This longitudinal data would further give the required facts to determine whether such mathematical changes are sensible forerunners of injury, causing a paradigm shift between the model being a descriptive model or even a predictive model that can be used to diagnose injuries.

## 5. Conclusions

This study developed a powerful two-dimensional analytical framework that integrates advanced EMG techniques with machine learning to analyze single-leg landings for injury risk prediction in Taekwondo athletes. Among the classification models tested, the RF model achieved the highest performance, with an F1 score of 0.8547, demonstrating the strong capability of ensemble learning to capture complex, non-linear muscle coordination patterns underlying stable landings.

In regression analysis, predicting muscle activation level has been used as an indicator of injury risk; the RR model showed an excellent fit with an R^2^ of 0.999. These near-perfect results show the thoughtful construction of functional features, including normalized mean, standard deviation, and activation ratio, which successfully linearized the relationship between EMG signals and injury risk, allowing the model to make highly accurate predictions.

At this point, the model is a highly precise descriptive technique of neuromuscular changes. Independent validation on more heterogeneous populations, such as female athletes and different levels of skills, is necessary to obtain true clinical utility. The integration of biomechanical measures of gold standard and prospective, longitudinal designs should be a future priority to confirm the correlation between these EMG measures and actual injury incidence. It is only after this validation that this framework can be effectively applied by the trainers and physicians as a proactive diagnostic tool for injury prevention.

## Figures and Tables

**Table 1:** Machine learning model’s configuration parameters.

Type	Model	Parameters
Classification	Logistic Regression	max_iter = 1000
	Random Forest Classifier	n_estimators = 200; random_state = 42
	XGBoost Classifier	n_estimators = 200; max_depth = 3; learning_rate = 0.1; eval_metric = ‘logloss’
	Voting Classifier	estimators = (LR, RF, XGB); voting = ‘soft’
Regression	Ridge Regression	alpha = 1.0
	Random Forest Regressor	n_estimators = 200; random_state = 42
	XGBoost Regressor	n_estimators = 200; max_depth = 3; learning_rate = 0.1; random_state = 42

**Table 2:** Performance evaluation metrics for classification results.

Model	Test Accuracy	F1-Score	5-Fold CV Accuracy (Mean ± Std)
Random Forest Classifier	0.8365	0.8547	0.7980 ± 0.0422
Voting Classifier	0.8269	0.8475	0.7763 ± 0.0367
XGB Classifier	0.7981	0.8174	0.7691 ± 0.0360
Logistic Regression	0.6731	0.7213	0.6729 ± 0.0488

**Table 3:** Performance evaluation metrics for regression models.

Model	Train R^2^ Score	Test R^2^ Score	Mean Absolute Error (MAE)	Root Mean Squared Error (RMSE)	5-Fold CV MAE (Mean ± Std Dev)
Ridge Regression	0.9999	0.9974	0.2620	0.4284	0.2459 ± 0.0270
XGB Regression	0.9997	0.9999	0.4302	0.8086	0.8268 ± 0.2604
Random Forest Regression	0.9982	0.9999	0.6260	2.1562	0.7302 ± 0.4616

**Table 4:** Comparison of the current study with related combat sports literature.

Study	Sport	Method	Model	Key Findings
[17]	Taekwondo	Impact vs. no-impact kicks	ANOVA	Neuromuscular control varies by skill; single-parameter approach
[27]	General athletics	Lower-limb movement recognition	Random Forest	High accuracy for discrete movement classification
[28]	Taekwondo	Ballistic movement EMG	Statistical comparison	Signal processing method critically affects EMG values in ballistic movements
[29]	Soccer	Prospective cohort study using preseason anthropometric and physical performance assessments.	XGBoost	Injury prediction achieved high precision
Current Study (2025)	Taekwondo	Single-leg landing classification	Random Forest, XGBoost	EMG patterns differentiate neuromuscular adaptations

## Data Availability

The datasets used and/or analyzed during the current study are available from the corresponding author upon reasonable request.

## Abbreviations

The following abbreviations are used in this manuscript:

EMG	Electromyography
AI	Artificial Intelligence
ACL	Anterior Cruciate Ligament
RMS	Root Mean Square
SMOTE	Synthetic Minority Oversampling Technique
LR	Logistic Regression
RF	Random Forest
XGBoost/XGB	eXtreme Gradient Boosting
RR	Ridge Regression
CV	Cross-Validation
MAE	Mean Absolute Error
RMSE	Root Mean Squared Error
R^2^	Coefficient of Determination
SHAP	SHapley Additive exPlanations
SENIAM	Surface ElectroMyoGraphy for the Non-Invasive Assessment of Muscles
